# Zoledronic acid impairs myeloid differentiation to tumour-associated macrophages in mesothelioma

**DOI:** 10.1038/sj.bjc.6605814

**Published:** 2010-07-27

**Authors:** J D Veltman, M E H Lambers, M van Nimwegen, R W Hendriks, H C Hoogsteden, J P J J Hegmans, J G J V Aerts

**Affiliations:** 1 Department of Pulmonary Medicine, Erasmus Medical Centre Rotterdam, PO Box 2040, Rotterdam 3000 CA, The Netherlands; 2 Departement of Pulmonary Diseases, Amphia Hospital, molengracht 21, Breda 4818CK, The Netherlands

**Keywords:** Zoledronic acid, myeloid differentiation, macrophages, myeloid-derived suppressor cells, malignant mesothelioma

## Abstract

**Background:**

Suppressive immune cells present in tumour microenvironments are known to augment tumour growth and hamper efficacy of antitumour therapies. The amino-bisphosphonate Zoledronic acid (ZA) is considered as an antitumour agent, as recent studies showed that ZA prolongs disease-free survival in cancer patients. The exact mechanism is a topic of debate; it has been suggested that ZA targets tumour-associated macrophages (TAMs).

**Methods:**

We investigate the role of ZA on the myeloid differentiation to TAMs in murine mesothelioma *in vivo* and *in vitro*. Mice were intraperitoneally inoculated with a lethal dose of mesothelioma tumour cells and treated with ZA to determine the effects on myeloid differentiation and survival.

**Results:**

We show that ZA impaired myeloid differentiation. Inhibition of myeloid differentiation led to a reduction in TAMs, but the number of immature myeloid cells with myeloid-derived suppressor cell (MDSC) characteristics was increased. In addition, ZA affects the phenotype of macrophages leading to reduced level of TAM-associated cytokines in the tumour microenvironment. No improvement of survival was observed.

**Conclusion:**

We conclude that ZA leads to a reduction in macrophages and impairs polarisation towards an M2 phenotype, but this was associated with an increase in the number of immature myeloid cells, which might diminish the effects of ZA on survival.

Recently, cancer-related inflammation was recognised as the seventh hallmark of cancer (Colotta *et al*, 2009). Infiltration of inflammatory cells into tumour tissues can be detected in many cancers. These infiltrating immune cells can possess immune stimulatory (antitumour activity) or immune suppressive capacity and thus promote tumour progression. In the tumour microenvironment of a progressing tumour, regulatory T cells, myeloid-derived suppressor cells (MDSCs) and tumour-associated macrophages (TAMs) have an important role in facilitating tumour growth and immune escape by suppressing antitumour effector cells (Bronte *et al*, 2001; Hegmans *et al*, 2006; Sakaguchi *et al*, 2008; Sica *et al*, 2008; Allavena *et al*, 2008b; Gabrilovich and Nagaraj, 2009).

Regulatory T cells and MDSCs are regarded as immune suppressive, as they are capable of inducing T-cell apoptosis and T-cell tolerance (Nagaraj and Gabrilovich, 2007; Sakaguchi *et al*, 2009), whereas TAMs are involved in a variety of processes, including angiogenesis, tumour invasion and tumour metastasis (Pollard, 2004; Condeelis and Pollard, 2006). Tumour-associated macrophages are derived from circulating monocytic precursors. Recently, it has been shown that this group of immature myeloid cells can be derived in three different populations, carrying MDSC characteristics, and can also function as a pool of precursor cells for macrophages and endothelial cells. Mononuclear-MDSC (MO-MDSC) harbours immune suppressive capacities, but additionally function as TAM precursors, in contrast to polymorph nuclear-MDSC (PMN-MDSC) and CD11b^high^Gr-1^low^-MDSC (Bronte and Zanovello, 2005; Nagaraj and Gabrilovich, 2007; Gabrilovich and Nagaraj, 2009; Greifenberg *et al*, 2009).

Tumour-associated macrophages acquire a polarised phenotype of alternatively activated macrophages (M2), whereas classically activated macrophages (M1) are more associated with inflammation. These two types of macrophages differ in receptor expression, effector function, and cytokine and chemokine productions (Mantovani *et al*, 2002). By producing pro-angiogenic enzymes, like matrix metalloproteinase 9 (MMP-9), TAMs are able to breakdown the extracellular matrix, leading to the release of pro-angiogenic proteins and growth factors. In addition, TAMs produce vascular endothelial growth factor (VEGF), promoting angiogenesis and recruitment of cells derived from the myeloid lineage (Kusmartsev and Gabrilovich, 2002; Siveen and Kuttan, 2009).

The recruitment of immature myeloid cells is enhanced by the production of chemokine (C–C motif) ligand-2/monocyte-chemotactic protein-1 (CCL-2/MCP-1), which is produced and expressed by TAMs and tumour cells. Within the heterogeneous group of immature myeloid cells, subpopulations are defined, which can further differentiate into mature macrophages, and hence TAMs provide their own precursor cells, which enhances tumour progression (Rossner *et al*, 2005; Umemura *et al*, 2008; Greifenberg *et al*, 2009; Dolcetti *et al*, 2010).

Targeting TAMs seems a promising tool to prevent tumour progression, thereby enhancing antitumour therapies. Bisphosphonates have been reported to target macrophages. They were initially prescribed to improve bone density; however, in metastasing cancers, a possible role in prevention of metastasis and prolongation of disease-free survival has been described (Diel *et al*, 2000, 2008; Gnant *et al*, 2009). The exact mechanisms by which bisphosphonates prevent disease progression are still a topic of extensive investigations. It has been proposed that bisphosphonates have a direct apoptotic effect on tumour cells (Yoneda *et al*, 2000; Mundy, 2001, 2002; Fromigue *et al*, 2003). In addition, indirect effects of bisphosphonates have been reported. It has been suggested that bisphosphonates modulate the immune response and may influence macrophage phenotype (Wolf *et al*, 2006; Tsagozis *et al*, 2008; Coscia *et al*, 2009).

Currently, we are investigating novel therapeutic strategies in mesothelioma, like dendritic cell-based immunotherapy. Mesothelioma is a cancer with dismal prognosis deriving from the layers of the pleural cavity or the peritoneal cavity. As high numbers of TAMs are found in pleural effusions and tumour biopsies in human mesothelioma, we determined the efficacy of a bisphosphonate, Zolendric acid (ZA) (Zometa; Novartis Pharma BV, Arnhem, The Netherlands), on TAM formation and tumour progression in a murine mesothelioma model.

In this study, we analysed the effect of ZA on myeloid differentiation both *in vitro* and *in vivo*. In addition, we determined whether ZA changed the phenotype of macrophages. Animal studies were performed to investigate the effect of ZA on survival.

## Methods

### Animals and cell lines

CBA-j mice (specific pathogen free, female, 6–8 weeks old) were purchased from Harlan (Huntington, UK) and were housed under pathogen-free conditions at the Erasmus MC animal facility. All experiments were approved by the local ethical committee for animal welfare (Erasmus University Committee of Animal Experts, Rotterdam, the Netherlands) and complied with the guidelines for the welfare of animals in experimental neoplasia by the United Kingdom Coordinating Committee on Cancer Research (UKCCCR) and by the Code of Practice of the Dutch Veterinarian Inspection. The AC29 cell line is kindly provided by Professor BWS Robinson (School of Medicine and Pharmacology, Sir Charles Gairdner Hospital Unit, The University of Western Australia, Perth, Australia).

### Depletion of macrophages *in vivo*

Macrophages were depleted by intraperitoneal (i.p.) injection of liposome-encapsulated clodronate (Dr Nico Van Rooijen, VUmc, FdG, Amsterdams, The Netherlands) (Claassen, 1992; Van Rooijen and Sanders, 1994).

CBA-j mice were i.p. inoculated with a lethal dose of 20 × 10^6^ AC29 tumour cells on day 0. Mice were i.p. injected with 200 *μ*l liposome-encapsulated clodronate or liposome-encapsulated PBS at days 5 and 10 after tumour injection. At day 12 mice were killed using CO_2_. The peritoneal cavity of tumour-bearing mice was washed with 1 ml PBS to obtain peritoneal cells for FACS analysis. All visible tumour material was excised from each mouse and data are expressed as wet weight (accuracy of 0.001 g).

Tumour-associated macrophages were defined by the expression of F4/80, MHCII and CD206 (marker for M2 macrophage phenotype).

### Immunohistochemistry on tumour biopsies

Tumour material was obtained 25 days after tumour injection from the peritoneal cavity of tumour-bearing mice. Tumour biopsies were embedded in Tissue-Tek II optimum cutting temperature medium (Miles, Naperville, IL, USA), snap frozen in liquid nitrogen and stored at −80°C. Tissue sections (6 *μ*m) were cut in an HM-560 cryostat (Microm, Heidelberg, Germany). F4/80 (Dr L Boon, Bioceros, Utrecht, The Netherlands) and CD206 (Serotec, Oxford, UK) primary antibodies were incubated for 1 h at room temperature. Binding of antibodies was detected using the immunoalkaline phosphatase antialkaline phosphatase method (DAKO, Glostrup, Denmark). Naphthol-AS-MX-phosphate (0.30 mg ml^−1^; Sigma-Aldrich Chemie BV, Zwijndrecht, The Netherlands) and new fuchsine (160 mg ml^−1^ in 2 M HCl; Chroma-Gesellschaft, Köngen, Germany) were used as substrate. The specificity of the antibodies was checked using a protein concentration-matched non-relevant monoclonal antibody and PBS.

### Flow cytometry

Spleens were aseptically removed and mechanically dispersed in cold PBS. Cell suspensions were filtered through a 100-*μ*m nylon cell strainer (BD Biosciences, Bedford, MA, USA), depleted of erythrocytes by osmotic shock, washed twice in RPMI and adjusted to a concentration of 1 × 10^6^ cells ml^−1^ in FACS buffer.

Splenocytes were stained with the following monoclonal antibodies: Ly6C (FITC), F4/80 (FITC), MHCII (PE), CD11c (PE-Texas red), CD11b (PerCP-Cy5.5), CD31 (PE-Cy7), CD206 (Alexa 647), Ly6G (Alexa Fluor 700), Gr-1 (APC-Cy7) and a fixable live/dead marker in DAPI (Invitrogen, Breda, The Netherlands). The final analysis and graphical output were performed using FlowJo software (Tree Star Inc., Costa Mesa, CA, USA).

### Macrophage culture

Single-cell suspensions of bone marrow, isolated from femurs and tibias of naïve mice, were cultured in RPMI supplemented with 10% foetal calf serum, 2.5 ml gentamicin (10 mg ml^−1^) (Gibco, Breda, the Netherlands) and *β*-mercaptoethanol (Sigma-Alderich) (further referred to as culture medium).

Bone marrow cells were isolated from the femurs and tibias of naïve mice under sterile conditions (Inaba *et al*, 1992). In short, all muscle tissues are removed with gauze from the bones and placed in a 60-mm dish with 70% alcohol for 1 min, washed twice with PBS and transferred into a fresh dish with RPMI 1640. Both ends of the bones were cut with scissors in the dish, and then the marrow was flushed out using 2 ml of RPMI 1640 with a syringe and 25-gauge needle. The tissue was suspended, passed through nylon mesh to remove small pieces of bone and debris, and red cells were lysed with ammonium chloride. A concentration of 10 ng ml^−1^ M-CSF (R&D systems, Oxon, UK) was used in our bone marrow cultures according to the protocol of Wan *et al* (2007, 2009)) or 30% tumour supernatant from AC29 cell culture (at 80% confluency) was added on day 0 to a culture of 2 × 10^6^ bone marrow-derived cells. AC29 was cultured in RPMI-1640 (Gibco, Paisley, UK) supplemented with GlutaMax, 10 mM HEPES and 5% heat-inactivated foetal bovine serum. When confluency reached 80% (typically 3–4 days), supernatant was collected, centrifuged for 10 min at 1000 **g** to remove cells/cell debris. Supernatant is added to the bone marrow culture at a concentration of 30%. This concentration was selected based on findings by Tsagozis *et al* (2008), who described that 30% tumour supernatant generated macrophages with a characteristic TAM phenotype. Zoledronic acid (ZA) (Zometa, Novartis Pharma BV) was added on day 0 to the culture conditions in different concentrations: 0.03, 0.15 or 0.3 *μ*M. All culture experiments have been repeated five times under comparable conditions.

Mononuclear-MDSCs were isolated from the spleen of tumour-bearing mice. Four to six colour samples were sorted using a FACSAria equipped with FACSDIVA software (BD Biosciences). After sorting, cells were cultured with M-CSF for 6 days. This experiment has been repeated twice under comparable conditions.

### Zoledronic acid treatment protocol

CBA-j mice were divided into two groups: each group consisting of 10 mice. On day 0, all mice were i.p. injected with a lethal dose of 20 × 10^6^ AC29 tumour cells. One group was treated daily with subcutaneous (s.c.) ZA injections (100 *μ*g kg^−1^, ∼2.5 *μ*g per mice) in 100 *μ*l PBS and the other group was treated with 100 *μ*l PBS as a control starting at day 5. This dosage was proven effective and non-toxic in mice (Stathopoulos *et al*, 2008).

Of both groups, five mice were killed at day 25. The others were killed when found profoundly ill. The occurrence of tumour growth, body weight, physical well-being and survival were measured for 2 months as described previously (Hegmans *et al*, 2005). Survival experiments were repeated three times under comparable conditions.

### Enzyme-linked immunosorbent assay

To measure cytokine levels, effusion fluid from the peritoneal cavity of tumour-bearing mice was obtained at day 25 after tumour injection. IL-10, IL-12, TNF*α*, IL-6, IL-1*β*, CCL-2 (MCP-1) and VEGF levels in the effusion fluid were determined using a specific ELISA assay (IL-10, IL-12 and TNF*α*: R&D systems, Abingdon, UK; IL-6, IL-1*β* and CCL-2 (MCP-1): BD Biosciences; VEGF: Calbiochem, Darmstadt, Germany). Protocols were followed as per the manufacturer's instructions. Samples were diluted appropriately to ensure that readings were within the limits of accurate detection. Results are expressed as pg ml^−1^ of effusion fluid.

### Statistical analysis

Data are expressed as mean±s.d. Comparisons between groups were made using *t*-tests. A two-tailed *P*-value <0.05 was considered significant. Data presented as a percentage of tumour-free animals were analysed with Kaplan–Meier survival curves, using the log-rank test to determine significance.

## Results

### Macrophages are essential in the onset and tumour development

We investigated the effect of the depletion of macrophages on tumour progression in a murine model for mesothelioma by treating mice with liposome-encapsulated clodronate. These liposomes are readily taken up by phagocytic cells, including macrophages, and induce cell-specific apoptosis after clodronate is set free into the cytoplasm of cells (Claassen, 1992; Van Rooijen and Sanders, 1994).

Mice were i.p. inoculated with a lethal dose of AC29 tumour cells and mice were treated with liposome-encapsulated clodronate or liposome-encapsulated PBS on days 5 and 10 after tumour injection. Mice were killed 12 days after tumour injection. All visible tumour material was excised from each mouse and wet weight was measured (accuracy of 0.001 g. FACS analysis was performed to verify the effectiveness of macrophage depletion (Mantovani *et al*, 2002; Sica *et al*, 2006).

**Figure 1:**
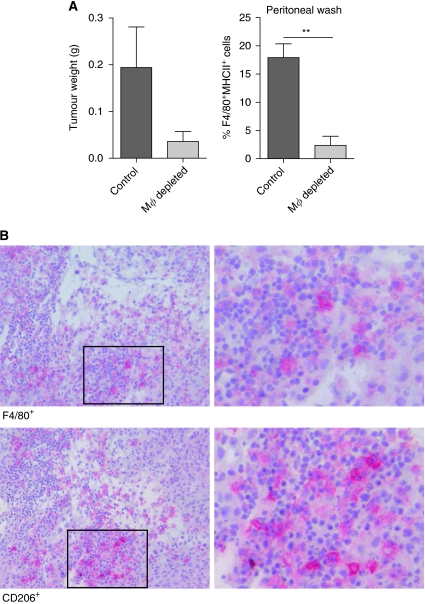
Depletion of macrophages inhibits tumour development. On day 1, mice were i.p. injected with a lethal dose of AC29 mesothelioma tumour cells and were treated twice with liposome-encapsulated clodronate (macrophage depletion) or liposome-encapsulated PBS (control) on days 5 and 10 after tumour injection (*n*=10). Twelve days after tumour injection, mice were killed and tumour weight was measured. All visible tumour material was excised from each mouse and data are expressed as wet weight (accuracy of 0.001 g). FACS analysis was performed to verify the effectiveness of macrophage depletion using liposome-encapsulated clodronate. Tumour biopsies were embedded in Tissue-Tek II and snap frozen in liquid nitrogen. Tissue sections (6 *μ*m) were analysed for the presence of macrophages. (**A**) Effect of macrophage depletion on tumour growth. Tumour growth was observed in all five mice treated with control liposomes; in contrast, only two out of five mice treated with macrophage-depleting liposomes developed visible tumour growth on day 12. Significant reduction in the percentage of F4/80^+^MHCII^+^ cells was found in the peritoneal cavity of macrophage-depleted mice (*P*=0.0015). Tumour weight was found to be lower in macrophage-depleted animals (*P*=0.077). (**B**) TAMs in murine mesothelioma. Tumour biopsies of control-treated mice showed infiltration of F4/80^+^ and CD206^+^ cells (magnification: upper, × 200; lower, × 400). ^**^P<0.001.

Treatment with liposome-encapsulated clodronate significantly reduced the number of macrophages in the peritoneal cavity of tumour inoculated mice (*P*=0.0015). All mice (*n*=5) treated with liposome-encapsulated PBS showed profound tumour growth at day 12. Three of the five mice treated with liposome-encapsulated clodronate had no visible tumour. In the case of mice that did develop tumour, tumour growth was less profound (Figure 1A). Macrophages (M1/M2) were found scattered throughout the tumour of a control mice (Figure 1B).

These data show that macrophages have a significant role in the onset and progression of tumour in our murine mesothelioma model.

### Zoledonic acid inhibits myeloid differentiation *in vitro*

To investigate the effect of ZA on myeloid differentiation *in vitro*, total bone marrow cells were cultured with M-CSF or 30% tumour supernatant in the presence or absence of ZA for 6 days using similar culture conditions as described by others (Wan *et al*, 2007, 2009; Tsagozis *et al*, 2008). FACS analysis was performed daily. ZA was added in different concentrations: 0.03, 0.15 or 0.30 *μ*M.

**Figure 2:**
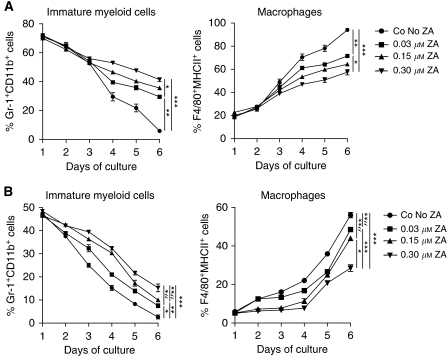
ZA inhibits differentiation of myeloid cells *in vitro*. The effect of ZA on myeloid differentiation was determined by cultured bone marrow-derived cells with 0.5 *μ*g ml^−1^ M-CSF or RPMI containing 30% tumour supernatant. Cells were cultured for 6 days. ZA was added to the cultures on day 0 in different concentrations (0.03, 0.15 or 0.30 *μ*M). FACS analysis was performed daily. ZA inhibits the downregulation of Gr-1^+^ cells and the upregulation of F4/80^+^ and MHCII^+^ cells in a dose-dependent manner. Experiments were repeated and data of five individual experiments were then combined. No significant differences were observed in the number of cells between the different culture conditions. A significant difference was found in the percentage of immature myeloid cells and the percentage of macrophages after 6 days of culture in both, resulting in a higher number of immature cells and a lower number of macrophages in ZA culture conditions. (**A**) Bone marrow culture with M-CSF: immature myeloid cells, ^*^P=0.004; ^**^P=0.0001; ^***^P<0.0001; macrophages, ^*^P=0.0003; ^**^P<0.0001; ^***^P=0.005. (**B**) Bone marrow culture with 30% tumour supernatant: immature myeloid cells, ^*^P=0.016; ^**^P=0.0014; ^***^P=0.0025; ^*^″*P*=0.015; ^**^″*P*=0.036; macrophages, ^*^P=0.002; ^**^P=0.0011; ^***^P=0.006; ^*^″*P*=0.0004, ^**^″*P*=0.001.

In both culture conditions there was a rapid decline in the number of cells expressing the immature marker Gr-1; however, the downregulation of Gr-1 was delayed when ZA was added to the culture. Simultaneously, a delay in the upregulation of macrophage markers was observed when ZA was added. The delay in the downregulation of Gr-1 and the upregulation of F4/80 and MHCII was dose dependent, leading to a reduced number of mature macrophages after 6 days of culture when ZA was added to the culture (Figure 2). The total number of cells within the different culture conditions did not significantly change over time.

To summarise, we observed an inhibition of myeloid differentiation to macrophages when ZA was added to the culture conditioned for macrophages. This inhibitory effect on differentiation was dose dependent and led to significant differences in the number of macrophages and immature cells between the different culture conditions on day 6. Furthermore, we showed that tumour-derived factors present in tumour supernatant induced the development of macrophages from bone marrow-derived cells.

### Zoledronic acid shifts the balance from M2 macrophages to M1 macrophages *in vitro*

To investigate the influence of ZA on the differentiation of myeloid progenitor cells to macrophage phenotypes, bone marrow cells were cultured in the presence or absence of 0.30 *μ*M ZA. Cells were cultured with M-CSF or 30% tumour supernatant as described previously (Wan *et al*, 2007, 2009; Tsagozis *et al*, 2008). The mannose receptor CD206 is a specific marker for M2 macrophages; therefore, the expression of this marker was analysed during culture to observe changes in macrophage phenotype (Mantovani *et al*, 2002).

**Figure 3:**
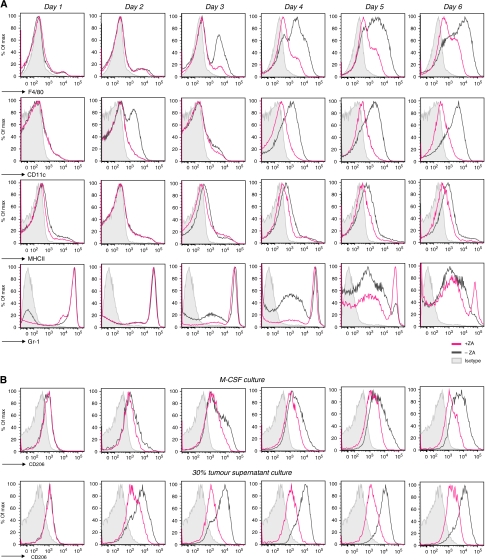
ZA inhibits the upregulation of extracellular markers *in vitro*. Expression profiles of M-CSF and RPMI containing 30% tumour supernatant cultured cells were measured by FACS to determine the effect of ZA addition to the culture (0.5 *μ*g ml^−1^ M-CSF; 0.30 *μ*M ZA was added on day 0). CD206 on macrophages was analysed to determine changes in macrophage phenotype. (**A**) M-CSF culture. F4/80, CD11c and MHCII were upregulated within 6 days. The immature myeloid marker Gr-1 was rapidly downregulated. The addition of ZA to the culture supernatant reduced the upregulation F4/80 and MHCII and CD11c, leading to a significant difference in MFI of these markers on day 6 of culture (*P*=0.003, 0.0023, 0.0003, respectively). As a consequence, the expression of Gr-1 was still high in a majority of the cells after 6 days of culture. (**B**) CD206 expression on macrophages (M-CSF culture and 30% tumour supernatant culture). After day 5, almost all F4/80^+^MHCII^+^ cells in the M-CSF culture expressed CD206. The upregulation of CD206 on cells cultured in the presence of tumour supernatant was more explicit. The addition of ZA to the cultures reduced the expression of CD206 on macrophages in both conditions and a significant reduction in the MFI of CD206 on macrophages after 6 days of culture (*P*<0.0001). Experiments were repeated several times under comparable conditions (*n*=5). Determination of the significance of peak shifts was based on calculation of the MFI.

Both M-CSF and 30% tumour supernatant culture conditions revealed that macrophage markers, F4/80, CD11c and MHCII, were rapidly upregulated within 6 days of culture. Simultaneously, the marker for immature myeloid cells Gr-1 was downregulated. However, the addition of ZA to the culture significantly inhibits the upregulation of F4/80, MHCII and especially CD11c, leading to a significant difference in mean fluorescent intensity (MFI) of these markers on day 6 of culture (*P*=0.003, 0.0023 and 0.0003, respectively). The expression of Gr-1 remained high in both these culture conditions (Figure 3A).

On day 1, cells expressed CD206 at low levels; however, at day 2 of culture, a slight increase was found in both conditions. This upregulation persisted in the following days in the M-CSF culture conditions. In those conditions where ZA was added to the M-CSF culture, the upregulation of CD206 was abolished. The upregulation of CD206 took place earlier in the tumour supernatant conditions compared with the M-CSF culture. As a consequence, the blocking effect of ZA on the generation of M2 macrophages was more profound, leading to a complete inhibition in the upregulation of CD206 by ZA and a significant reduction in the MFI of CD206 on macrophages after 6 days of culture (*P*<0.0001) (Figure 3B).

In conclusion, these data showed that the addition of ZA to macrophage-inducing culture conditions significantly inhibits the upregulation of F4/80, MHCII and CD11c. In addition, these data reveal that addition of tumour supernatant leads to polarisation of the macrophage phenotype towards M2, and that ZA can prevent this polarisation *in vitro*, leading to a significant reduction in the MFI of CD206 on macrophages cultured in the presence of ZA (*P*<0.0001).

### Zoledronic acid does not improve survival in a murine tumour model

Next, we investigated whether treatment with ZA had an effect on survival. Mice were i.p. inoculated with AC29 tumour cells and treated daily with s.c. ZA injections or PBS as a control. The dosing schedule was chosen according to the literature, in which it was proven effective and non-toxic (Stathopoulos *et al*, 2008). Mice were weighted on a daily basis and killed when found profoundly ill. The effusion fluid was collected when mice were killed.

**Figure 4:**
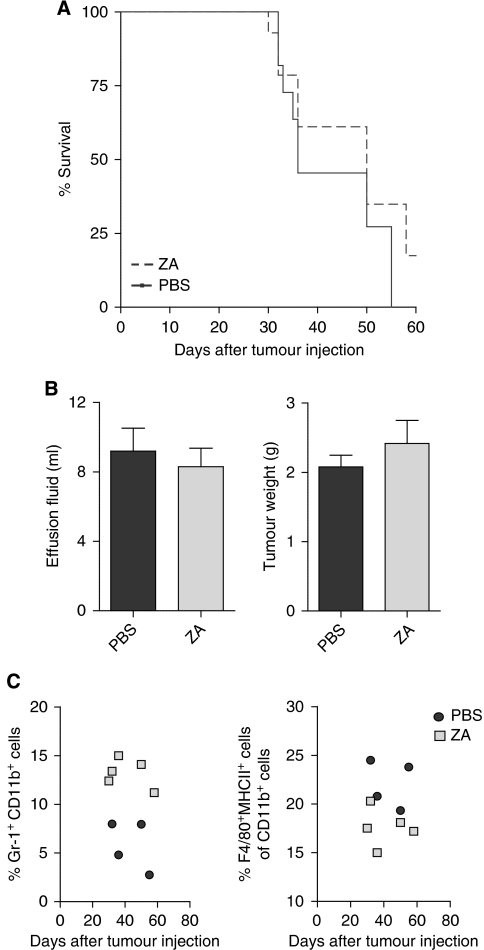
Treatment with ZA does not improve survival. Mice were divided into two groups (*n*=10 mice per group). Mice were treated daily with s.c. ZA (100 *μ*g kg^−1^, ∼2.5 *μ*g per mice) or PBS injections starting on day 5 after tumour injection. This dosing schedule was proven effective and non-toxic (Stathopoulos *et al*, 2008). Mice were killed when found profoundly ill. No significant improvement of survival was measured. (**A**) Kaplan–Meier survival curve. No significant differences in survival were observed between mice treated daily with s.c. injection of ZA compared with untreated mice (*P*=0.3675). (**B**) Malignant effusions and tumour weights. Tumour weight and the amount of malignant effusion were measured when mice were killed. The effusion fluid was removed from the peritoneal cavity by fine-needle aspiration and all visible tumour material was collected. No significant differences in tumour weight or the amount of malignant effusion were observed (*P*=0.42 and 0.61). (**C**) Myeloid cell types in the spleen of tumour-bearing mice. Long-term treatment effects were observed in the number of myeloid cells within the spleen of tumour-bearing mice, implicating that higher numbers of myeloid precursors and lower numbers of TAMs were detected in mice treated with ZA compared with untreated mice.

No significant prolongation of survival was observed upon treatment with ZA (*P*=0.2183) (Figure 4A). No significant differences were found in the total body weight or in the amount of effusion fluid volume during the experiment (*P*=0.4178 and 0.6103, respectively) (Figure 4B). Long-term treatment effects showed that higher numbers of myeloid precursors and lower numbers of TAMs were detected in mice treated with ZA compared with untreated mice; however, no effects were found in tumour burden and survival (Figure 4C).

Taken together, although no significant differences on tumour progression and survival could be observed between untreated mice and mice treated with ZA, a reduction in the number of macrophages and an increase in the number of immature myeloid cells was detected.

### Identification of myeloid cells *in vivo*

To establish whether ZA inhibits myeloid differentiation in tumour-bearing mice, myeloid cell types within the spleen, tumour and peritoneal cavity of tumour-bearing mice were identified. Recently, it has been shown that the heterogeneous group of MDSC consists of three major groups: polymorph nuclei CD11b^high^Gr-1^high^ MDSC (PMN-MDSC), mononuclear CD11b^high^Gr-1^int^ MDSC (MO-MDSC) and the CD11b^high^Gr-1^low^-MDSC (Greifenberg *et al*, 2009). Identification of MDSC subtypes was carried out on cells of untreated tumour-bearing mice 25 days after tumour inoculation. The population of PMN-MDSC produces high levels of reactive oxygen species leading to the downregulation of the *ζ*-chain of T cells, resulting in T-cell tolerance, whereas MO-MDSC can directly inhibit T-cell expansion by the production of nitric oxide-inducing T-cell apoptosis (Bronte and Zanovello, 2005; Nagaraj and Gabrilovich, 2007; Gabrilovich and Nagaraj, 2009).

**Figure 5:**
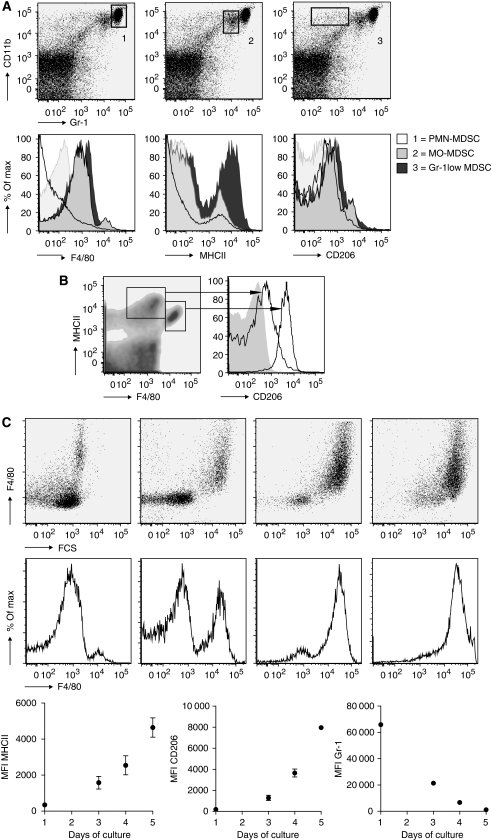
Identification of myeloid cells in tumour-bearing mice. To identify the effect of tumour growth on the recruitment of myeloid cells during tumour progression, mice were inoculated with tumour cells and killed on day 25 (*n*=12). (**A**) Identification of myeloid cell types in splenocytes of tumour-bearing mice. Immature myeloid cells could be divided into three groups, as described in the literature (Greifenberg *et al*, 2009). The Gr-1^low^-MDSC showed intermediate expression of F4/80, MHCII and CD206. F4/80 expression was found in MO-MDSC, but not in PMN-MDSC. A small number of MO-MDSC expressed high levels of F4/80 MHCII and CD206. MHCII and CD206 expressions were low in PMN-MDSC. (**B**) Identification of type I and type II macrophages in splenocytes of tumour-bearing mice. Two populations of macrophages could be identified; a high expression of CD206 was found in the membrane of the population with a high expression of F4/80 but lower expression of MHCII. (**C**) M-CSF culture of MO-MDSC-sorted cell fraction. MO-MDSC were sorted from splenocytes of tumour-bearing mice and cultured with 0.5 *μ*g ml^−1^ M-CSF. The expression of F4/80, MHCII, CD206 and Gr-1 were measured on consecutive days.

A massive increase in MDSC was found in the spleens and effusion fluids from the peritoneal cavity of tumour-bearing mice on day 25 after tumour injection. MDSCs were further subdivided into three groups: PMN-MDSC, MO-MDSC and Gr-1^low^-MDSC. All subgroups of MDSC stained intermediate positive for the marker CD206 (Figure 5A). Expression of the markers F4/80 and MHCII was found on MO-MDSC and Gr-1^low^-MDSC, but not on PMN-MDSC. A small subpopulation of the MO-MDSC was strongly positive for F4/80, MHCII and CD206 (Figure 5A).

In the peritoneal cavity and in the spleen, M1 and M2 macrophages were identified based on the expression of F4/80, MHCII and CD206, and forward–sideward scatter patron. M2 macrophages express higher levels of CD206 and F4/80 and lower levels of MHCII compared with the M1 macrophage population (Figure 5B).

New insights have revealed that MO-MDSC are pluripotent and under certain conditions are able to differentiate into TAMs (Rossner *et al*, 2005; Umemura *et al*, 2008; Greifenberg *et al*, 2009; Dolcetti *et al*, 2010). This is also reflected *in vivo*, as the population of MO-MDSC already showed partial expression of F4/80 and MHCII in an early stage compared with PMN-MDSC. To confirm that MO-MDSC could further differentiate into macrophages, we sorted this population from the spleen of tumour-bearing mice and cultured the cells for 5 days in the presence of M-CSF. Mononuclear-MDSC upregulated the expression of F4/80 and MHCII rapidly, in contrast to the PMN-MDSC that were not able to upregulate F4/80 under the influence of M-CSF (Figure 5C).

To summarise, we were able to identify three populations of MDSC as described in the literature in our tumour model. The MO-MDSCs were found to be able to differentiate into macrophages. Two populations of macrophages based on CD206 expression were found both in the spleen and in the peritoneal cavity of tumour-bearing mice.

### Zoledronic acid reduces macrophages but increases the number of immature myeloid cells during tumour development

As we observed a difference in the number of macrophages and immature myeloid cells during ZA treatment, we next wanted to know whether ZA treatment also affects macrophage phenotype *in vivo*. To investigate whether ZA influences the development of macrophages during tumour progression *in vivo*, mice were i.p. inoculated with AC29 tumour cells and treated daily with s.c. ZA injections (100 *μ*g kg^−1^, ∼2.5 *μ*g per mice) or PBS as a control. Mice were killed 25 days after tumour injection.

**Figure 6:**
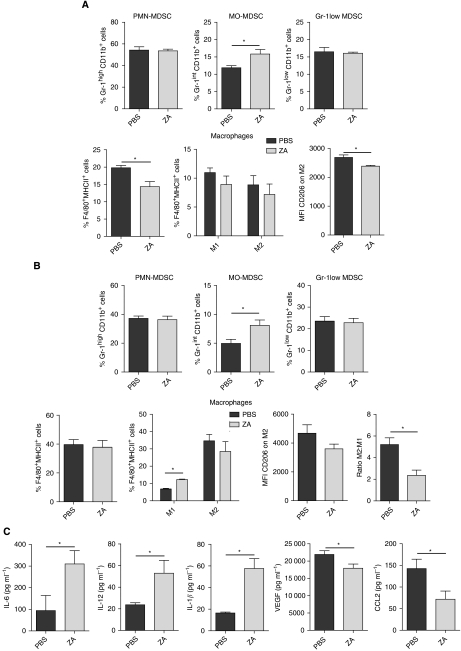
ZA changes M1:M2 ratio and increases MO-MDSC in tumour-bearing mice. Mice were i.p. inoculated with AC29 tumour cells and treated daily with s.c. injection of ZA. (100 *μ*g kg^−1^) or PBS as a control (*n*=6 each group). Mice were killed 25 days after tumour injection. The number of MDSC was analysed according to the subdivision as described in Figure 5. Macrophages were subdivided into M1 and M2 macrophages based on the co-expression of CD206, F4/80 and MHCII on the membrane. (**A**) Myeloid cell types in spleen of tumour-bearing mice. MO-MDSC were significantly increased in the spleen of ZA-treated animals (^*^P=0.0312). No difference was found in the percentage of PMN-MDSC and Gr-1^low^-MDSC (^*^P=0.77 and 0.75). The percentage of total macrophages in the spleen of ZA-treated mice was significantly lower compared with untreated mice (^*^P=0.0091). In the spleen of tumour-bearing mice, although not significant there was a trend towards a reduction in both M1 and M2 macrophages in ZA-treated mice. In addition, ZA treatment significantly lowers the MFI of CD206 on M2 macrophages (^*^P=0.0095). (**B**) Myeloid cell types in effusion fluid of tumour-bearing mice. MO-MDSCs were significantly increased in the effusion fluid of ZA-treated animals (^*^P=0.034). No difference was found in the percentage of PMN-MDSC and Gr-1^low^-MDSC or macrophages (^*^P=0.72 and 0.74). A significant increase in M1 macrophages was found (^*^P=0.035), and also an increase was found in the number of M2 macrophages (^*^P=0.33). ZA shifts the balance, leading to a significant difference in the ratio of M1:M2 macrophages (^*^P=0.011); a trend towards a lower MFI of CD206 was observed (^*^P=0.114). (**C**) Cytokines in effusion fluid of tumour-bearing mice. ELISA was performed on effusion fluid of tumour-bearing mice treated with ZA or PBS as a control. A significant increase in IL-6, IL-12 and IL-1*β* was found in ZA-treated mice (^*^P=0.049, 0.042 and 0.005, respectively). A significant reduction in VEGF and CCL-2 (MCP-1) expressions was found in ZA-treated mice (^*^P=0.05 and 0.039).

An increase in CD11b^+^Gr-1^+^ cells was found in the spleen and effusion fluid of tumour-bearing mice of both groups. A significant increase was found in MO-MDSC both in the spleen and in the effusion fluid from the peritoneal cavity (spleen: *P*=0.0312; effusion fluid: *P*=0.034), whereas no significant differences were found in the proportion of PMN-MDSC and Gr-1^low^-MDSC (spleen: *P*=0.77 and 0.75; effusion fluid: *P*=0.74 and 0.72). The proportion of macrophages in the spleen of ZA-treated mice was significantly lower compared with PBS-treated mice (*P*=0.0091). There was a trend towards a shift in macrophage phenotype from M2 to M1; however, the reduction in M2 macrophages was not significant (*P*=0.18). However, we found that the expression levels of CD206, expressed as the MFI, were significantly lower on M2 macrophages of ZA-treated mice compared with M2 macrophages in the spleen of PBS-treated mice (*P*=0.0095) (Figure 6A). No significant decrease was found in the percentage of macrophages in the effusion fluid of ZA-treated mice (*P*=0.75). However, a significant increase in M1 macrophages was found, while M2 macrophages were reduced (*P*=0.035 and 0.33). A shift from M2 macrophage to M1 macrophage phenotype was found based on the MFI expression of CD206, confirming the findings in the spleen (*P*=0.14). As a result, a difference in ratio of M1:M2 was found (*P*=0.011) (Figure 6B).

Enzyme-linked immunosorbent assay on the effusion fluid was performed to determine whether the reduction on CD206 expression on macrophages in ZA-treated mice was accompanied by changes in cytokine, chemokine and growth factor production. A significant increase was found in the levels of IL-6, IL-12 and IL-1*β* in the effusion fluid of ZA-treated mice (*P*=0.049, 0.042 and 0.005, respectively). In addition, we observed a significant reduction in VEGF and CCL-2 (MCP-1) in the effusion fluid of ZA-treated animals (*P*=0.05 and 0.039). Levels of IL-10 and TNF*α* were not detectable in the effusion fluid.

In conclusion, we have shown that treatment with ZA reduces the number of macrophages, but at the same time, we observed higher levels of immature myeloid cell types. When we further defined the population of immature myeloid cells, significantly more MO-MDSC were found. In addition, we found that the expression of CD206 on macrophages was lower in ZA-treated animals. This reduced expression of the M2 macrophages marker was accompanied with a significant reduction in VEGF and CCL-2 (MCP-1) levels and a significant increase in the levels of IL-6, IL-12 and IL-1*β* (Figure 6C).

## Discussion

Many effects of bisphosphonates have been reported in literature. Treatment with ZA prolongs the 1-year survival rate in breast cancer, prostate cancer and bladder cancer (Gnant, 2009; Rajpar *et al*, 2010; Zaghloul *et al*, 2010). However, exact mechanisms by which ZA prevents disease progression is still a topic of investigation. The role of bone formation and mineralisation in relation to adhesion and tumour outgrowth in these structures has gained interest after treatment with bisphosphonates appear to have protective effects on the prevention of bone metastasis (Boissier *et al*, 1997, 2000; Magnetto *et al*, 1999; Montague *et al*, 2004). Besides the reduction in bone metastasis, studies have shown that ZA also prevents metastasis to secondary organs (Hiraga *et al*, 2004). Therefore, other mechanisms, during tumour progression, may be influenced by ZA treatment. Recently, it has been suggested that ZA may work as immune modulator and may therefore be applicable as and antitumour agent (Clezardin, 2005; Tsagozis *et al*, 2008; Santini *et al*, 2010).

ZA is an aminobisphosphonate that targets the mevalonate pathway in myeloid cells (Wolf *et al*, 2006). Recently, several clinical trails have been performed to investigate the role of ZA in tumour progression (Saad *et al*, 2002, 2004; Gainford *et al*, 2005). It has been suggested that ZA has direct antineoplastic activity on tumour cells (Santini *et al*, 2003; Clezardin, 2005; Ohtsuka *et al*, 2005; Caraglia *et al*, 2007). However, other studies have shown that clinically observed effects of ZA treatment may also be explained by indirect mechanisms involving immune modulation.

Studies have shown that ZA eventually leads to stimulation and proliferation of gamma/delta T cells (Kunzmann *et al*, 2000; Miyagawa *et al*, 2001; Sato *et al*, 2005). Besides, direct effects of ZA on tumour cell have been reported in breast cancer, prostate and bladder cancer (Gnant *et al*, 2009; Rajpar *et al*, 2010; Zaghloul *et al*, 2010). However, more recently the effect of ZA on macrophages has gained interest. Macrophages are known to have a dominant role within the tumour microenvironment. Several studies have reported the importance of TAMs on tumour progression (Pollard, 2004; Sica *et al*, 2006, 2008; Sangaletti *et al*, 2008; Allavena *et al*, 2008b; Coffelt *et al*, 2009). We and others have shown that depleting TAMs with liposome-encapsulated clodronate inhibits tumour growth and prolongs survival (Zeisberger *et al*, 2006; Miselis *et al*, 2008).

As our *in vitro* data revealed that ZA inhibits the differentiation towards macrophages, our aim was to determine whether this inhibition in differentiation also led to a reduction in TAMs *in vivo*.

The percentage of macrophages in the spleen was significantly reduced in ZA-treated animals compared with the control group. In the effusion fluid, no significant difference was found in the total number of macrophages; however, a shift towards a more M1 macrophage phenotype was observed. Recent studies have shown that TAMs can be derived from certain subpopulations within the heterogeneous MDSC population (Rossner *et al*, 2005; Umemura *et al*, 2008; Greifenberg *et al*, 2009; Dolcetti *et al*, 2010). From these studies, it has been established that TAMs were mainly derived from the MO-MDSC population. A significant increase in MO-MDSC was found both in the spleen and in the effusion fluid of mice treated with ZA. As we found that ZA inhibits myeloid differentiation *in vitro*, it can be assumed that the significant increase in the MO-MDSC population found in ZA-treated mice compared with untreated mice are caused by similar inhibitory effects on myeloid differentiation. Inhibition of myeloid differentiation by ZA has also been reported by others (Wolf *et al*, 2006; Melani *et al*, 2007; Chen *et al*, 2009).

Tsagozis *et al* (2008) reported that ZA shifts TAMs from an M2-like phenotype to an M1-like phenotype, resulting in a reduction in TAM-associated cytokine production *in vitro*. We were able to confirm these data *in vitro*, but were also able to show that these changes also occur *in vivo*. A reduction in the M2-associated receptor (CD206) was found on macrophages in tumour-bearing mice treated with ZA. Changes in M2-associated cytokines, chemokines and growth factors were observed in the effusion fluid after ZA treatment. We showed that ZA lowered the levels of VEGF in the effusion fluid of tumour-bearing mice. The reduction in VEGF and MMP-9 expressions by macrophages under the influence of ZA has been described to prevent tumour neovascularisation (Santini *et al*, 2002; Giraudo *et al*, 2004; Vincenzi *et al*, 2005; Melani *et al*, 2007; Tsagozis *et al*, 2008). The production of MMPs and VEGF is known to be essential during tumour progression and especially facilitating metastases (Allavena *et al*, 2008b; Coffelt *et al*, 2009).

Even though it was found that ZA inhibits myeloid differentiation and shifts the balance from M2 macrophage phenotype towards an M1 macrophage phenotype, this did not improve survival in our model. There are several explanations for this finding. One of the most striking findings was the increased levels of immature myeloid cells (MDSC) due to treatment with ZA. Recently, studies have reported the role of MDSC in tumour progression. MDSC are most known for their immune suppressive function and induction of tumour-specific T-cell tolerance facilitating tumour immune escape (Bronte and Zanovello, 2005; Nagaraj and Gabrilovich, 2007; Gabrilovich and Nagaraj, 2009). Therefore, the inhibition of myeloid development may lead to reduction in the number of TAMs, resulting in a higher number of MDSC, which also promote tumour progression.

A second reason as to why ZA has no effect on survival may be assigned to the shift in macrophage phenotype. As most studies showed that TAMs are polarised to an M2 phenotype that contribute to carcinogenesis, shifting the phenotype of macrophages to M1 seems promising. M1 macrophages produce less tumour growth-promoting soluble factors and can potentially kill tumour cells. However, the question rises whether shifting the phenotype in full-blown tumours is possible and whether repolarisation is as promising as believed previously (Mills *et al*, 1992; Mantovani *et al*, 2002; Weigert and Brune, 2008). Negative aspects of iNOS+ M1 macrophages were also observed. Actually, these cells can kill tumour cells, but as a consequence pro-carcinogenic substances are released from dying cells, leading to enhanced angiogenesis and enhanced polarisation of M1 macrophages to an M2 phenotype (Weigert and Brune, 2008). Coscia *et al.* reported similar observation. In their study, repolarisation of the macrophage phenotype was most promising during early tumour development, resulting in prolonged tumour-free survival and overall survival. In addition, they notify that shifting the phenotype in a host with a large tumour burden can be hazardous and no effects of ZA administration were observed when *in situ* carcinomas had progressed. From their data, it appears that tumour development is inhibited when the induction of M2 macrophages can be post-poned. We observed similar findings: shifting the phenotype from M2 to M1 at later stages of tumourigenesis seems less effective. Therefore, it can be hypothesised that preventing the formation of M2 macrophages is a critical key in preventing tumour outgrowth during early stages of disease development.

A third explanation for the limited effect on survival could be that ZA has effects on processes during tumour development that do not have a dominant role in our tumour models. Most investigations are carried out in tumours with high metastasis rates in combination with other therapeutic approaches (like adjuvant endocrine therapy) (Saad *et al*, 2002; Hiraga *et al*, 2004; Gnant, 2009). In these tumours, metastasis may be the cause of death, in contrast to our model. As TAMs do have an essential role in metastasis, ZA may be beneficial in these types of tumours (Allavena *et al*, 2008a). However, in both human studies and animal models, survival is only marginally influenced by ZA, and also in tumours with high metastases rates (Hiraga *et al*, 2004; Stathopoulos *et al*, 2008; Pandya *et al*, 2010; Ottewell *et al*, 2010).

To study the effects of ZA on macrophages in the primary tumour, dose levels are higher than clinically advised. Doses selected for clinical trials were based on changes in bone resorption markers (including serum COOH-terminal telopeptide and urinary *N*-telopeptide/creatinine ratio) and preliminary evidence of efficacy on skeletal-related events; however, no studies were performed comparing dose–effect relations on the tumour microenvironment. Although there are some concerns on side effects caused by ZA at higher levels (Ibrahim *et al*, 2003), additional studies are required to determine the safety and efficacy of increased dosages for this new application.

In conclusion, our data show that daily administration of ZA in tumour-bearing mice inhibits myeloid differentiation and shifts the balance from M2 macrophage phenotype to M1 macrophage phenotype. However, as a consequence the number of immature myeloid cells remains high in the spleen and in the effusion fluid, especially in the MO-MDSC population. This MO-MDSC population was found to have an immunosuppressive effect to which we ascribe the fact that survival was not improved with ZA in our study.

## References

[bib1] Allavena P, Sica A, Garlanda C, Mantovani A (2008a) The Yin–Yang of tumor-associated macrophages in neoplastic progression and immune surveillance. Immunol Rev 222: 155–161 18364000 10.1111/j.1600-065X.2008.00607.x

[bib2] Allavena P, Sica A, Solinas G, Porta C, Mantovani A (2008b) The inflammatory micro-environment in tumor progression: the role of tumor-associated macrophages. Crit Rev Oncol Hematol 66: 1–9 17913510 10.1016/j.critrevonc.2007.07.004

[bib3] Boissier S, Ferreras M, Peyruchaud O, Magnetto S, Ebetino FH, Colombel M, Delmas P, Delaisse JM, Clezardin P (2000) Bisphosphonates inhibit breast and prostate carcinoma cell invasion, an early event in the formation of bone metastases. Cancer Res 60: 2949–2954 10850442

[bib4] Boissier S, Magnetto S, Frappart L, Cuzin B, Ebetino FH, Delmas PD, Clezardin P (1997) Bisphosphonates inhibit prostate and breast carcinoma cell adhesion to unmineralized and mineralized bone extracellular matrices. Cancer Res 57: 3890–3894 9307266

[bib5] Bronte V, Serafini P, Apolloni E, Zanovello P (2001) Tumor-induced immune dysfunctions caused by myeloid suppressor cells. J Immunother 24: 431–446 11759067 10.1097/00002371-200111000-00001

[bib6] Bronte V, Zanovello P (2005) Regulation of immune responses by L-arginine metabolism. Nat Rev Immunol 5: 641–654 16056256 10.1038/nri1668

[bib7] Caraglia M, Marra M, Leonetti C, Meo G, D'Alessandro AM, Baldi A, Santini D, Tonini G, Bertieri R, Zupi G, Budillon A, Abbruzzese A (2007) R115777 (Zarnestra)/Zoledronic acid (Zometa) cooperation on inhibition of prostate cancer proliferation is paralleled by Erk/Akt inactivation and reduced Bcl-2 and bad phosphorylation. J Cell Physiol 211: 533–543 17192846 10.1002/jcp.20960

[bib8] Chen YJ, Chao KS, Yang YC, Hsu ML, Lin CP, Chen YY (2009) Zoledronic acid, an aminobisphosphonate, modulates differentiation and maturation of human dendritic cells. Immunopharmacol Immunotoxicol 31: 499–508 19555208 10.1080/08923970902814103

[bib9] Claassen E (1992) Detection, localization and kinetics of immunomodulating liposomes *in vivo*. Res Immunol 143: 235–241 1574653 10.1016/s0923-2494(92)80173-i

[bib10] Clezardin P (2005) Anti-tumour activity of zoledronic acid. Cancer Treat Rev 31(Suppl 3): 1–8 10.1016/j.ctrv.2005.09.002 16225995

[bib11] Coffelt SB, Hughes R, Lewis CE (2009) Tumor-associated macrophages: effectors of angiogenesis and tumor progression. Biochim Biophys Acta 1796(1): 11–18 19269310 10.1016/j.bbcan.2009.02.004

[bib12] Colotta F, Allavena P, Sica A, Garlanda C, Mantovani A (2009) Cancer-related inflammation, the seventh hallmark of cancer: links to genetic instability. Carcinogenesis 30: 1073–1081 19468060 10.1093/carcin/bgp127

[bib13] Condeelis J, Pollard JW (2006) Macrophages: obligate partners for tumor cell migration, invasion, and metastasis. Cell 124: 263–266 16439202 10.1016/j.cell.2006.01.007

[bib14] Coscia M, Quaglino E, Iezzi M, Curcio C, Pantaleoni F, Riganti C, Holen I, Monkkonen H, Boccadoro M, Forni G, Musiani P, Bosia A, Cavallo F, Massaia M (2009) Zoledronic acid repolarizes tumor-associated macrophages and inhibits mammary carcinogenesis by targeting the mevalonate pathway. J Cell Mol Med (accepted for publication) 10.1111/j.1582-4934.2009.00926.x PMC3822730 19818098

[bib15] Diel IJ, Jaschke A, Solomayer EF, Gollan C, Bastert G, Sohn C, Schuetz F (2008) Adjuvant oral clodronate improves the overall survival of primary breast cancer patients with micrometastases to the bone marrow: a long-term follow-up. Ann Oncol 19: 2007–2011 18664560 10.1093/annonc/mdn429 PMC2733118

[bib16] Diel IJ, Solomayer EF, Bastert G (2000) Bisphosphonates and the prevention of metastasis: first evidences from preclinical and clinical studies. Cancer 88: 3080–3088 10898355 10.1002/1097-0142(20000615)88:12+<3080::aid-cncr27>3.0.co;2-w

[bib17] Dolcetti L, Peranzoni E, Ugel S, Marigo I, Fernandez Gomez A, Mesa C, Geilich M, Winkels G, Traggiai E, Casati A, Grassi F, Bronte V (2010) Hierarchy of immunosuppressive strength among myeloid-derived suppressor cell subsets is determined by GM-CSF. Eur J Immunol 40: 22–35 19941314 10.1002/eji.200939903

[bib18] Fromigue O, Kheddoumi N, Body JJ (2003) Bisphosphonates antagonise bone growth factors' effects on human breast cancer cells survival. Br J Cancer 89: 178–184 12838321 10.1038/sj.bjc.6601009 PMC2394205

[bib19] Gabrilovich DI, Nagaraj S (2009) Myeloid-derived suppressor cells as regulators of the immune system. Nat Rev Immunol 9: 162–174 19197294 10.1038/nri2506 PMC2828349

[bib20] Gainford MC, Dranitsaris G, Clemons M (2005) Recent developments in bisphosphonates for patients with metastatic breast cancer. BMJ 330: 769–773 15802719 10.1136/bmj.330.7494.769 PMC555882

[bib21] Giraudo E, Inoue M, Hanahan D (2004) An amino-bisphosphonate targets MMP-9-expressing macrophages and angiogenesis to impair cervical carcinogenesis. J Clin Invest 114: 623–633 15343380 10.1172/JCI22087 PMC514591

[bib22] Gnant M (2009) Bisphosphonates in the prevention of disease recurrence: current results and ongoing trials. Curr Cancer Drug Targets 9: 824–833 20025570 10.2174/156800909789760267

[bib23] Gnant M, Mlineritsch B, Schippinger W, Luschin-Ebengreuth G, Postlberger S, Menzel C, Jakesz R, Seifert M, Hubalek M, Bjelic-Radisic V, Samonigg H, Tausch C, Eidtmann H, Steger G, Kwasny W, Dubsky P, Fridrik M, Fitzal F, Stierer M, Rucklinger E, Greil R, Investigators A-T, Marth C (2009) Endocrine therapy plus zoledronic acid in premenopausal breast cancer. N Engl J Med 360: 679–691 19213681 10.1056/NEJMoa0806285

[bib24] Greifenberg V, Ribechini E, Rossner S, Lutz MB (2009) Myeloid-derived suppressor cell activation by combined LPS and IFN-gamma treatment impairs DC development. Eur J Immunol 39: 2865–2876 19637228 10.1002/eji.200939486

[bib25] Hegmans JP, Hemmes A, Aerts JG, Hoogsteden HC, Lambrecht BN (2005) Immunotherapy of murine malignant mesothelioma using tumor lysate-pulsed dendritic cells. Am J Respir Crit Care Med 171: 1168–1177 15764728 10.1164/rccm.200501-057OC

[bib26] Hegmans JP, Hemmes A, Hammad H, Boon L, Hoogsteden HC, Lambrecht BN (2006) Mesothelioma environment comprises cytokines and T-regulatory cells that suppress immune responses. Eur Respir J 27: 1086–1095 16540497 10.1183/09031936.06.00135305

[bib27] Hiraga T, Williams PJ, Ueda A, Tamura D, Yoneda T (2004) Zoledronic acid inhibits visceral metastases in the 4T1/luc mouse breast cancer model. Clin Cancer Res 10: 4559–4567 15240548 10.1158/1078-0432.CCR-03-0325

[bib28] Ibrahim A, Scher N, Williams G, Sridhara R, Li N, Chen G, Leighton J, Booth B, Gobburu JV, Rahman A, Hsieh Y, Wood R, Vause D, Pazdur R (2003) Approval summary for zoledronic acid for treatment of multiple myeloma and cancer bone metastases. Clin Cancer Res 9: 2394–2399 12855610

[bib29] Inaba K, Inaba M, Romani N, Aya H, Deguchi M, Ikehara S, Muramatsu S, Steinman RM (1992) Generation of large numbers of dendritic cells from mouse bone marrow cultures supplemented with granulocyte/macrophage colony-stimulating factor. J Exp Med 176: 1693–1702 1460426 10.1084/jem.176.6.1693 PMC2119469

[bib30] Kunzmann V, Bauer E, Feurle J, Weissinger F, Tony HP, Wilhelm M (2000) Stimulation of gammadelta T cells by aminobisphosphonates and induction of antiplasma cell activity in multiple myeloma. Blood 96: 384–392 10887096

[bib31] Kusmartsev S, Gabrilovich DI (2002) Immature myeloid cells and cancer-associated immune suppression. Cancer Immunol Immunother 51: 293–298 12111117 10.1007/s00262-002-0280-8 PMC11034227

[bib32] Magnetto S, Boissier S, Delmas PD, Clezardin P (1999) Additive antitumor activities of taxoids in combination with the bisphosphonate ibandronate against invasion and adhesion of human breast carcinoma cells to bone. Int J Cancer 83: 263–269 10471537 10.1002/(sici)1097-0215(19991008)83:2<263::aid-ijc19>3.0.co;2-t

[bib33] Mantovani A, Sozzani S, Locati M, Allavena P, Sica A (2002) Macrophage polarization: tumor-associated macrophages as a paradigm for polarized M2 mononuclear phagocytes. Trends Immunol 23: 549–555 12401408 10.1016/s1471-4906(02)02302-5

[bib34] Melani C, Sangaletti S, Barazzetta FM, Werb Z, Colombo MP (2007) Amino-biphosphonate-mediated MMP-9 inhibition breaks the tumor-bone marrow axis responsible for myeloid-derived suppressor cell expansion and macrophage infiltration in tumor stroma. Cancer Res 67: 11438–11446 18056472 10.1158/0008-5472.CAN-07-1882 PMC2646404

[bib35] Mills CD, Shearer J, Evans R, Caldwell MD (1992) Macrophage arginine metabolism and the inhibition or stimulation of cancer. J Immunol 149: 2709–2714 1401910

[bib36] Miselis NR, Wu ZJ, Van Rooijen N, Kane AB (2008) Targeting tumor-associated macrophages in an orthotopic murine model of diffuse malignant mesothelioma. Mol Cancer Ther 7: 788–799 18375821 10.1158/1535-7163.MCT-07-0579

[bib37] Miyagawa F, Tanaka Y, Yamashita S, Minato N (2001) Essential requirement of antigen presentation by monocyte lineage cells for the activation of primary human gamma delta T cells by aminobisphosphonate antigen. J Immunol 166: 5508–5514 11313389 10.4049/jimmunol.166.9.5508

[bib38] Montague R, Hart CA, George NJ, Ramani VA, Brown MD, Clarke NW (2004) Differential inhibition of invasion and proliferation by bisphosphonates: anti-metastatic potential of Zoledronic acid in prostate cancer. Eur Urol 46: 389–401; discussion 401–2 15306113 10.1016/j.eururo.2004.04.022

[bib39] Mundy G (2001) Preclinical models of bone metastases. Semin Oncol 28: 2–8 10.1016/s0093-7754(01)90225-8 11544569

[bib40] Mundy GR (2002) Metastasis to bone: causes, consequences and therapeutic opportunities. Nat Rev Cancer 2: 584–593 12154351 10.1038/nrc867

[bib41] Nagaraj S, Gabrilovich DI (2007) Myeloid-derived suppressor cells. Adv Exp Med Biol 601: 213–223 17713008 10.1007/978-0-387-72005-0_22

[bib42] Ohtsuka Y, Manabe A, Kawasaki H, Hasegawa D, Zaike Y, Watanabe S, Tanizawa T, Nakahata T, Tsuji K (2005) RAS-blocking bisphosphonate zoledronic acid inhibits the abnormal proliferation and differentiation of juvenile myelomonocytic leukemia cells *in vitro*. Blood 106: 3134–3141 16046524 10.1182/blood-2005-03-0972

[bib43] Ottewell PD, Lefley DV, Cross SS, Evans CA, Coleman RE, Holen I (2010) Sustained inhibition of tumor growth prolonged survival following sequential administration of doxorubicin zoledronic acid in a breast cancer model. Int J Cancer 126: 522–532 19621384 10.1002/ijc.24756

[bib44] Pandya KJ, Gajra A, Warsi GM, Argonza-Aviles E, Ericson SG, Wozniak AJ (2010) Multicenter, randomized, phase 2 study of zoledronic acid in combination with docetaxel and carboplatin in patients with unresectable stage IIIB or stage IV non-small cell lung cancer. Lung Cancer 67(3): 330–338 19493585 10.1016/j.lungcan.2009.04.020

[bib45] Pollard JW (2004) Tumour-educated macrophages promote tumour progression and metastasis. Nat Rev Cancer 4: 71–78 14708027 10.1038/nrc1256

[bib46] Rajpar S, Massard C, Laplanche A, Tournay E, Gross-Goupil M, Loriot Y, Di Palma M, Bossi A, Escudier B, Chauchereau A, Fizazi K (2010) Urinary N-telopeptide (uNTx) is an independent prognostic factor for overall survival in patients with bone metastases from castration-resistant prostate cancer. Ann Oncol (in press) 10.1093/annonc/mdq037 20181574

[bib47] Rossner S, Voigtlander C, Wiethe C, Hanig J, Seifarth C, Lutz MB (2005) Myeloid dendritic cell precursors generated from bone marrow suppress T cell responses via cell contact and nitric oxide production *in vitro*. Eur J Immunol 35: 3533–3544 16331707 10.1002/eji.200526172

[bib48] Saad F, Gleason DM, Murray R, Tchekmedyian S, Venner P, Lacombe L, Chin JL, Vinholes JJ, Goas JA, Chen B (2002) A randomized, placebo-controlled trial of zoledronic acid in patients with hormone-refractory metastatic prostate carcinoma. J Natl Cancer Inst 94: 1458–1468 12359855 10.1093/jnci/94.19.1458

[bib49] Saad F, Gleason DM, Murray R, Tchekmedyian S, Venner P, Lacombe L, Chin JL, Vinholes JJ, Goas JA, Zheng M (2004) Long-term efficacy of zoledronic acid for the prevention of skeletal complications in patients with metastatic hormone-refractory prostate cancer. J Natl Cancer Inst 96: 879–882 15173273 10.1093/jnci/djh141

[bib50] Sakaguchi S, Wing K, Onishi Y, Prieto-Martin P, Yamaguchi T (2009) Regulatory T cells: how do they suppress immune responses? Int Immunol 21: 1105–1111 19737784 10.1093/intimm/dxp095

[bib51] Sakaguchi S, Yamaguchi T, Nomura T, Ono M (2008) Regulatory T cells and immune tolerance. Cell 133: 775–787 18510923 10.1016/j.cell.2008.05.009

[bib52] Sangaletti S, Di Carlo E, Gariboldi S, Miotti S, Cappetti B, Parenza M, Rumio C, Brekken RA, Chiodoni C, Colombo MP (2008) Macrophage-derived SPARC bridges tumor cell-extracellular matrix interactions toward metastasis. Cancer Res 68: 9050–9059 18974151 10.1158/0008-5472.CAN-08-1327

[bib53] Santini D, Vespasiani Gentilucci U, Vincenzi B, Picardi A, Vasaturo F, La Cesa A, Onori N, Scarpa S, Tonini G (2003) The antineoplastic role of bisphosphonates: from basic research to clinical evidence. Ann Oncol 14: 1468–1476 14504045 10.1093/annonc/mdg401

[bib54] Santini D, Vincenzi B, Avvisati G, Dicuonzo G, Battistoni F, Gavasci M, Salerno A, Denaro V, Tonini G (2002) Pamidronate induces modifications of circulating angiogenetic factors in cancer patients. Clin Cancer Res 8: 1080–1084 12006522

[bib55] Santini D, Virzi V, Fratto ME, Bertoldo F, Sabbatini R, Berardi R, Calipari N, Ottaviani D, Ibrahim T (2010) Can we consider Zoledronic acid a new antitumor agent? Recent evidence in clinical setting. Curr Cancer Drug Targets 10(1): 46–54 20088792 10.2174/156800910790980223

[bib56] Sato K, Kimura S, Segawa H, Yokota A, Matsumoto S, Kuroda J, Nogawa M, Yuasa T, Kiyono Y, Wada H, Maekawa T (2005) Cytotoxic effects of gammadelta T cells expanded *ex vivo* by a third generation bisphosphonate for cancer immunotherapy. Int J Cancer 116: 94–99 15756684 10.1002/ijc.20987

[bib57] Sica A, Larghi P, Mancino A, Rubino L, Porta C, Totaro MG, Rimoldi M, Biswas SK, Allavena P, Mantovani A (2008) Macrophage polarization in tumour progression. Semin Cancer Biol 18: 349–355 18467122 10.1016/j.semcancer.2008.03.004

[bib58] Sica A, Schioppa T, Mantovani A, Allavena P (2006) Tumour-associated macrophages are a distinct M2 polarised population promoting tumour progression: potential targets of anti-cancer therapy. Eur J Cancer 42: 717–727 16520032 10.1016/j.ejca.2006.01.003

[bib59] Siveen KS, Kuttan G (2009) Role of macrophages in tumour progression. Immunol Lett 123: 97–102 19428556 10.1016/j.imlet.2009.02.011

[bib60] Stathopoulos GT, Moschos C, Loutrari H, Kollintza A, Psallidas I, Karabela S, Magkouta S, Zhou Z, Papiris SA, Roussos C, Kalomenidis I (2008) Zoledronic acid is effective against experimental malignant pleural effusion. Am J Respir Crit Care Med 178: 50–59 18388351 10.1164/rccm.200710-1513OC

[bib61] Tsagozis P, Eriksson F, Pisa P (2008) Zoledronic acid modulates antitumoral responses of prostate cancer-tumor associated macrophages. Cancer Immunol Immunother 57: 1451–1459 18297280 10.1007/s00262-008-0482-9 PMC11030129

[bib62] Umemura N, Saio M, Suwa T, Kitoh Y, Bai J, Nonaka K, Ouyang GF, Okada M, Balazs M, Adany R, Shibata T, Takami T (2008) Tumor-infiltrating myeloid-derived suppressor cells are pleiotropic-inflamed monocytes/macrophages that bear M1- and M2-type characteristics. J Leukocyte Biol 83: 1136–1144 18285406 10.1189/jlb.0907611

[bib63] Van Rooijen N, Sanders A (1994) Liposome mediated depletion of macrophages: mechanism of action, preparation of liposomes and applications. J Immunol Methods 174: 83–93 8083541 10.1016/0022-1759(94)90012-4

[bib64] Vincenzi B, Santini D, Dicuonzo G, Battistoni F, Gavasci M, La Cesa A, Grilli C, Virzi V, Gasparro S, Rocci L, Tonini G (2005) Zoledronic acid-related angiogenesis modifications and survival in advanced breast cancer patients. J Interferon Cytokine Res 25: 144–151 15767788 10.1089/jir.2005.25.144

[bib65] Wan H, Coppens JM, van Helden-Meeuwsen CG, Leenen PJ, van Rooijen N, Khan NA, Kiekens RC, Benner R, Versnel MA (2009) Chorionic gonadotropin alleviates thioglycollate-induced peritonitis by affecting macrophage function. J Leukocyte Biol 86: 361–370 19414540 10.1189/jlb.0208126

[bib66] Wan H, Versnel MA, Cheung WY, Leenen PJ, Khan NA, Benner R, Kiekens RC (2007) Chorionic gonadotropin can enhance innate immunity by stimulating macrophage function. J Leukocyte Biol 82: 926–933 17626151 10.1189/jlb.0207092

[bib67] Weigert A, Brune B (2008) Nitric oxide, apoptosis and macrophage polarization during tumor progression. Nitric Oxide 19: 95–102 18486631 10.1016/j.niox.2008.04.021

[bib68] Wolf AM, Rumpold H, Tilg H, Gastl G, Gunsilius E, Wolf D (2006) The effect of zoledronic acid on the function and differentiation of myeloid cells. Haematologica 91: 1165–1171 16956814

[bib69] Yoneda T, Michigami T, Yi B, Williams PJ, Niewolna M, Hiraga T (2000) Actions of bisphosphonate on bone metastasis in animal models of breast carcinoma. Cancer 88: 2979–2988 10898341 10.1002/1097-0142(20000615)88:12+<2979::aid-cncr13>3.0.co;2-u

[bib70] Zaghloul MS, Boutrus R, El-Hossieny H, Kader YA, El-Attar I, Nazmy M (2010) A prospective, randomized, placebo-controlled trial of zoledronic acid in bony metastatic bladder cancer. Int J Clin Oncol (in press) 10.1007/s10147-010-0074-5 20354750

[bib71] Zeisberger SM, Odermatt B, Marty C, Zehnder-Fjallman AH, Ballmer-Hofer K, Schwendener RA (2006) Clodronate-liposome-mediated depletion of tumour-associated macrophages: a new and highly effective antiangiogenic therapy approach. Br J Cancer 95: 272–281 16832418 10.1038/sj.bjc.6603240 PMC2360657

